# Medication of Hydroxychloroquine, Remdesivir and Convalescent Plasma during the COVID-19 Pandemic in Germany—An Ethical Analysis

**DOI:** 10.3390/ijerph18115685

**Published:** 2021-05-26

**Authors:** Katja Voit, Cristian Timmermann, Florian Steger

**Affiliations:** Institute of the History, Philosophy and Ethics of Medicine, Ulm University, 89073 Ulm, Germany; cristian.timmermann@uni-ulm.de (C.T.); florian.steger@uni-ulm.de (F.S.)

**Keywords:** SARS-CoV-2, clinical ethics, pharmaceutical preparations, public health, epidemic, disease outbreak

## Abstract

This paper aims to analyze the ethical challenges in experimental drug use during the early stage of the COVID-19 pandemic, using Germany as a case study. In Germany uniform ethical guidelines were available early on nationwide, which was considered as desirable by other states to reduce uncertainties and convey a message of unity. The purpose of this ethical analysis is to assist the preparation of future guidelines on the use of medicines during public health emergencies. The use of hydroxychloroquine, remdesivir and COVID-19 convalescent plasma in clinical settings was analyzed from the perspective of the ethical principles of beneficence, non-maleficence, justice and autonomy. We observed that drug safety and drug distribution during the pandemic affects all four ethical principles. We therefore recommend to establish ethical guidelines (i) to discuss experimental treatment options with patients from all population groups who are in urgent need, (ii) to facilitate the recording of patient reactions to drugs in off-label use, (iii) to expand inclusion criteria for clinical studies to avoid missing potentially negative effects on excluded groups, and (iv) to maintain sufficient access to repurposed drugs for patients with prior conditions.

## 1. Introduction

The COVID-19 pandemic (Coronavirus disease 2019) has brought discussions on medical ethics to the foreground. In the last decades, medical ethics was mostly advanced on a theoretical basis in Germany, without the expectation of needing to provide immediate guidelines to urgent public health matters. From the first weeks in which the COVID-19 outbreak developed into a pandemic, medical ethics needed to suddenly make a substantial contribution to solving questions of fair distribution of limited medical goods such as protective equipment, intensive care beds and ventilators [1,2]. Moreover, in Germany, ethicists responded quickly with a series of recommendations. Noteworthy examples are the recommendations for regulating access to the COVID-19 vaccine with the participation of the German Ethics Council [3] and the advisory role of the Working Group of Medical Ethics Commissions in Germany on clinical research in the pandemic [4]. The Competence Network Public Health on COVID-19 has provided numerous position papers on the public health ethics dimensions of health policy decisions, including work on the ethical dimension of uncertainty [5] and tracing apps [6]. In relation to biomedical research with human subjects, the PRECOPE projects offers assistance in meeting the World Health Organization requirements for the transfer of existing ethical standards to the most important ethical research challenges on COVID-19 [7]. However, neither specialist societies nor relevant guidelines deal in detail with ethical questions relating the use of potential COVID-19 therapeutics during the pandemic. Since hydroxychloroquine and remdesivir have received the greatest attention during the initial phase of the pandemic, we focus on these two drugs in our article. In addition, we also include COVID-19 convalescent plasma as a third therapeutic approach to the two antiviral therapies. These three treatment options are already studied well enough to carry out a preliminary ethical analysis on experimental drug use during the pandemic.

The aim of our ethical analysis is to identify on the basis of these three early treatment options the potential ethical challenges in the use of experimental drugs during the first phase of the COVID-19 pandemic. For this purpose, central themes were identified in a thematic analysis of publications in scientific journals, ethics working groups and governmental institutions. These publications are analyzed using the four ethical principles according to Beauchamp and Childress [8]. Our main findings are discussed in relation to German health regulatory decisions. Germany was selected because ethics groups provided early on ethical guidelines, the work between ethics groups and public institutions was generally perceived as constructive, and the relevant publications were well received. Although it is unclear what impact ethical guidelines actually had on clinical practice, Germany managed to overcome the first COVID-19 wave with one of the lowest mortality rates in the region [9], which makes it an important case to explore. The knowledge gained on ethical challenges can help to prepare for comparable public health emergencies in the future.

## 2. Materials and Methods

In our ethical analysis, we began with a literature search to identify the main themes that require an ethical evaluation. As a first step, a systematic literature search was carried out on PubMed, Embase and Cochrane Library (including results from 1 January 2020 until 22 October 2020). The search terms included “COVID-19”, “ethics” and “drug use”. The purpose was to identify publications that address ethical aspects of drug use during the first ten months of the COVID-19 pandemic. Relevant publications were selected from the search results obtained after reviewing the titles and abstracts. The decisive factor for inclusion was the use of hydroxychloroquine, remdesivir or COVID-19 convalescent plasma to treat patients suffering from COVID-19 symptoms. Publications dealing with general ethical issues related to off-label use or compassionate use [10] during the COVID-19 pandemic were also included. Publications on studies that tracked the prophylactic administration of one of these drugs to study participants without SARS-CoV-2 infection were excluded. In a second step, we carried out a thematic analysis [11] of all included publications. We looked for relevant initial themes that touched on at least one of the four principles according to Beauchamp and Childress (respect for the patient’s autonomy, non-maleficence, beneficence and justice) [8]. The principles did not have to be named explicitly. If, for example, the exclusion of HIV-infected people from clinical studies was mentioned, this was viewed as significant due to the principle of respect for the patient’s autonomy. As a third step, by combining recurring initial themes and sub-themes, we inductively generated two main themes. We related the themes to the four ethical principles to reach new findings and draft our recommendations. Beauchamp and Childress’s four-principles-approach focuses primarily on individual cases and was not conceived for issues of collective well-being. However, particularly in the context of justice, the authors also refer to national and global health policy and the right to health care [8]. Furthermore, much of the off-label use focusses on individual patients, and in some cases, physicians assume substantial risks to save with unproven methods their patients. In our analysis, each of the four principles could be interpreted, and conflicts with the four principles were identified. A complete moral picture could be gathered.

## 3. Results of the Ethical Analysis

The literature search with the terms “COVID-19”, “ethics” and “drug use” led to *n* = 148 results (122 PubMed, 10 Embase, 16 Cochrane Library) for the first ten months of the year 2020. After removing duplications and publications in languages other than German or English, we were left with *n* = 140 publications. Based on the title or abstract, *n* = 88 results were excluded because they had drugs other than hydroxychloroquine, remdesivir or COVID-19 convalescent plasma as a central topic. *N* = 52 publications were searched for ethical themes. In these publications, we identified 19 initial themes, which we summarized into five relevant sub-themes:

1. Existing data on pharmacological treatment;
2. Drug research;
3. Treatment outside of clinical trials;
4. Non-medical influences on drug use (e.g., political or economic interests);
5. Distribution of limited medicinal products (including plasma).

**Figure 1:**
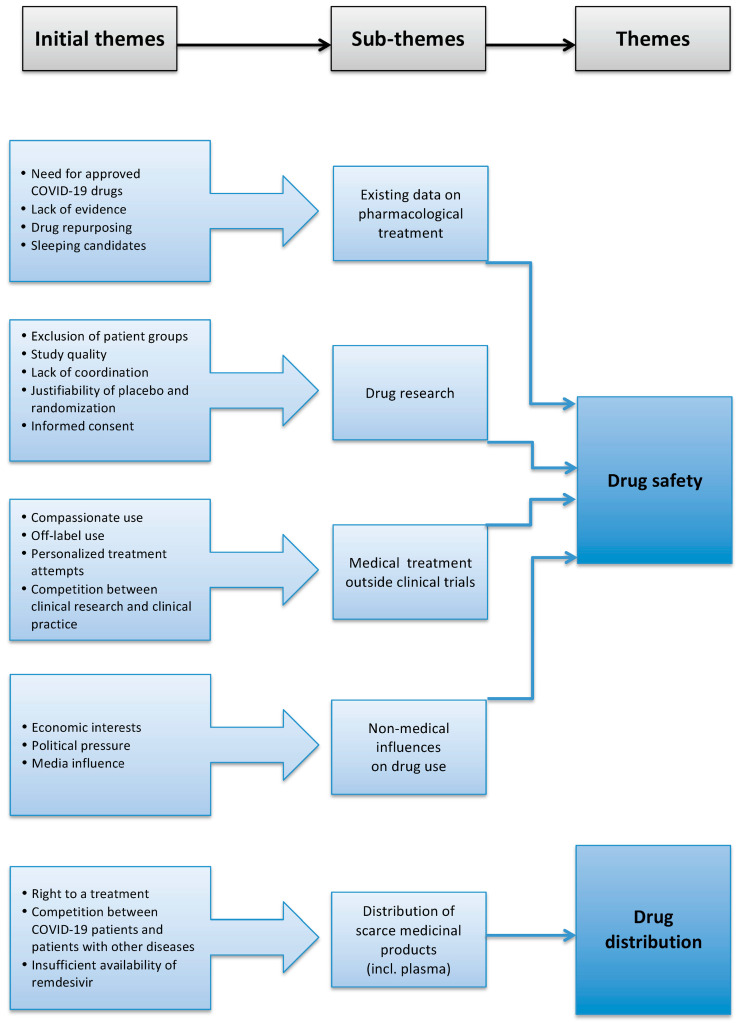
Development of the two main themes “drug safety” and “drug distribution” from five sub-themes formed out of 19 initial themes.

We combined the sub-themes 1 to 4 to form the theme “drug safety”. As the second main theme, we identified “drug distribution” (Figure 1). We related the identified ethical challenges to one or more of the four ethical principles according to Beauchamp and Childress.

Since COVID-19 is a new disease, and SARS-CoV-2 (Severe Acute Respiratory Syndrome Coronavirus 2) is a new pathogen, there were no approved and therefore tested drugs at the beginning of the pandemic. Several drugs that had already been approved for other diseases were evaluated. In addition, so-called sleeping candidates were tested. These are drugs that have been abandoned during the development process because they have proven to be insufficiently effective for the originally intended medical application or have unwanted side effects [12]. Drugs were used experimentally and were gradually granted drug approval, deviating from standard procedure. The drug approval process and the legal nature of drug use varies internationally (Box 1). For example, hydroxychloroquine, remdesivir and COVID-19 convalescent plasma had an emergency use authorization in the United States [13,14,15]. In other countries, these drugs have been given off-label uses or compassionate uses. Furthermore, from a legal perspective, terms such as emergency use, off-label use or compassionate use do not mean exactly the same thing in all countries.

Box 1 **Glossary of the main legal categories permitting the use of COVID-19 drugs.**

Regular marketing authorizationEuropean Union: The marketing authorization procedure evaluates whether a medicinal product is efficacious and safe and whether it has the required pharmaceutical quality [16].United States: FDA approval of a drug indicates that data on the drug’s effects have been reviewed and that the drug is determined to provide benefits that outweigh its known and potential risks for the intended patient group [17].Emergency useEuropean Union: EU member states can temporarily allow the marketing of an unauthorized medicinal product in emergency situations. Member states can decide that the requirements are less stringent than for a conditional marketing authorization [18].United States: The FDA may authorize unapproved medical products or unapproved uses during public health emergencies when no adequate and approved alternatives are available [19].Conditional marketing authorizationThis type of authorization is valid for one year and is awarded after an initial validation showing greater benefits than risks, on the condition that the new data is submitted as it becomes available [18].Off-label useThis is the use of an approved drug for purposes for which it had not originally received regulatory approval [20].Compassionate useEuropean Union: Compassionate use is the use of an unapproved drug that is given to a group of patients who have a disease that would result in severe disability or is life-threatening and cannot be satisfactorily treated with an approved drug [10].United States: Also called “expanded access”. It is an option for patients to get access to experimental drugs to treat life-threatening or serious health issues outside clinical trials when no satisfactory alternative treatment options are available [21].Necessity as a justification for useIn German law § 34 StGB is often used to exculpate physicians who attempt to save lives through non-approved methods, including non-approved drugs, as long as they are reasonable to avert danger [22].

### 3.1. Principle of Beneficence

The principle of beneficence demands from the practitioner to promote the well-being of the patient [8]. A connection to the principle of beneficence could be found for four of our five identified sub-themes: existing data on pharmacological treatment, drug research, treatment outside of clinical trials, and distribution of limited medicinal products (including plasma). Indications that the use of chloroquine, or hydroxychloroquine, can have potential benefits in COVID-19 patients were found in *n* = 24 (46.2%) of the publications analyzed. Hydroxychloroquine is listed in the class of antimalarials with antiviral immunomodulatory effects [23,24]. An effect could be demonstrated with SARS-CoV in vitro and in vivo, as well as with SARS-CoV-2 in vitro [23,24,25]. The safety profile of hydroxychloroquine is known from decades of use under other indications (malaria, rheumatic diseases) [26]. Numerous clinical studies are currently being carried out to investigate the effectiveness of hydroxychloroquine in COVID-19 [27]. Early results from clinical studies on hydroxychloroquine in COVID-19 patients have contradictory conclusions [24]. *N* = 15 (28.9%) publications mentioned a possible benefit of using remdesivir. Remdesivir is a nucleotide prodrug [23]. It is believed to inhibit the viral RNA-dependent RNA polymerase [28]. A decreased virus replication was detected for MERS-CoV (Middle East Respiratory Syndrome Coronavirus) and the Ebola virus in animal experiments [29]. Shorter disease manifestation has been reported among COVID-19 patients [28]. The treatment option with COVID-19 convalescent plasma was discussed in *n* = 8 (15.4%) of the analyzed publications. During therapy with convalescent plasma, the plasma of recovered patients is transfused with neutralizing antibodies to support the immune system [30]. Convalescent plasma has been administered in the past for Ebola, SARS-CoV, MERS-CoV and influenza [31]. The use of hydroxychloroquine, remdesivir or COVID-19 convalescent plasma in clinical studies has had scientific value and advantages for study participants. Through their participation, participants have the possibility to gain early access to treatments for which a therapeutic effect is expected [32]. Randomization and the administration of placebo may hinder direct benefits. This was often interpreted as a violation of the medical duty of beneficence. Failure to provide timely access has been suggested as a reason for deviating from established study designs during the pandemic [27]. Weaker evidence is accepted when this allows us to offer life-saving therapy more quickly [12]. The use of drugs in off-label use and compassionate use follows the principle of beneficence and does not primarily serve scientific purposes, such as gaining knowledge about the effectiveness of drugs [27,33]. In the absence of proven effective measures, off-label use can be in line with the Declaration of Helsinki, provided that patients received expert advice and informed consent was obtained [34]. Close coordination with ethics committees is recommended [33].

### 3.2. Principle of Non-Maleficence

The principle of non-maleficence demands avoiding interventions with harmful consequences [8]. This principle has implications on all five sub-themes. Recommendations for the medication of hydroxychloroquine, remdesivir or COVID-19 convalescent plasma to treat COVID-19 are mainly based on in vitro studies, results from animal experiments and experience with other viral infections [23,31]. Positive results from in vitro research or animal experiments cannot simply be transferred to humans [35]—even in emergency situations, the major physiological differences between species need to be kept in mind. The efficacy and safety of hydroxychloroquine for COVID-19 are not yet known. Cardiac side effects, for example, have been described for its use under other indications (e.g., malaria, rheumatic diseases) [26]. Studies with hydroxychloroquine as a treatment option were terminated due to emerging publications of studies that did not confirm any benefit for treating COVID-19 [36]. Remdesivir was originally developed for hepatitis C and was later considered for Ebola. However, remdesivir was not approved for the treatment of hepatitis C or Ebola because the drug did not meet efficacy requirements [35]. The drug can be described as a sleeping candidate [12]. The emergence of the new disease was a sudden unexpected opportunity to finally give remdesivir a major use. The identification of new uses for existing but abandoned drugs that previously did not generate profit is highly advantageous for pharmaceutical companies. Therefore, the sub-theme non-medical influences on drug use, in particular economic interests, also played a role. In the United States, remdesivir obtained emergency use authorization on 1 May 2020, based on preliminary study results [37]. The actual benefit of remdesivir is so far an open question [15], as the drug has not gone through the usual safety and efficacy review process that is normally required before a drug is approved.

An emergency use authorization does not correspond to the regular approval process of the drug authorities. The emergency use authorization is based on two clinical trials, one of them was conducted by the manufacturer. The results of both studies were not published at the time of the emergency use authorization [38]. A critical review process of the data by independent researchers to complement the analysis of the regulatory authority could not take place at the time of authorization. Such time-saving procedures come at the cost of transparency. In this respect, there are parallels to oseltamivir. The drug was used for treatment during the H1N1 influenza in 2009 outbreak based on limited research funded primarily by the drug company. Independent researchers who wanted to review the data from clinical trials were not granted access. Years later, it was proven that the drug did not have the effect promised by the manufacturer [39]. Earlier ethical guidelines on pandemic planning place for such reasons a high value on “openness & transparency” [40]. Independent review allows to identify adverse reactions which manufacturers may not fully disclose due to conflicts of interests. Furthermore, independent reviews allow to keep expectations realistic, so that patients and populations at risk are less likely to develop false hopes.

There are positive study results for the administration of convalescent plasma in COVID-19. However, the study participants received antiviral drugs at the same time. It could not be determined with certainty which effect was indeed triggered by COVID-19 convalescent plasma [30]. Most of the currently registered studies on COVID-19 convalescent plasma are not double-blind randomized clinical studies [41]. The inclusion of participants in studies that do not produce valid results is viewed as ethically unacceptable [42]. Possible risks of participating in the study are, among others, unknown serious side effects or an ineffective treatment [32]. The often standardized exclusion criteria for potential participants also comes with ethical conflicts. In particular, children, pregnant women, breastfeeding women, the elderly or people with comorbidities are underrepresented in medical research, which impedes adequate efficacy studies and the early identification of negative side effects. An automatic exclusion of groups that are underrepresented in medical research contradicts the Declaration of Helsinki, according to which these groups should have adequate access to medical research [34]. COVID-19 research has failed to include pregnant women, breastfeeding women, HIV-infected people, children and the elderly [23,43,44]. These groups may suffer harm from such exclusionary policies when results of studies performed on other groups cannot simply be translated to them [44,45,46]. Since hydroxychloroquine has already been used successfully for many years on pregnant women and children, the exclusion of these patient groups in COVID-19 studies is viewed as unjustified [44,45,46]. The exclusion of older COVID-19 patients has particularly harmful consequences, as it leads to study results that are not representative for the patient group with the highest COVID-19 mortality rates [43]. As a result, many patients have received hydroxychloroquine, remdesivir and COVID-19 convalescent plasma in risky off-label or compassionate use [24,47]. Even during emergencies, the use of drugs with poor evidence for off-label use can violate the principle of non-maleficence [33]. Drugs such as remdesivir, which are used in compassionate use programs, have not been used in large patient populations—serious side effects may therefore be unknown to date [47]. When using drugs outside clinical studies in compassionate use, there is no systematic evaluation of their efficacy [27].

### 3.3. Principle of Respect for Autonomy

Three of the five sub-themes relate to the principle of respect for autonomy: drug research, treatment outside of clinical trials, and distribution of limited medicinal products (including plasma). According to the principle of respect for autonomy, the patient’s informed consent is required before any therapeutic measures [8]. A careful informative process is a prerequisite for informed consent, which should not be waived or downplayed during a pandemic [12]. The fact that a drug has not received regular approval must be disclosed and patients need to be impartially informed about the possible risk and benefits involved in the use of such drugs. In the case of patients capable of giving consent, time constraints and the risk of confusing patients with the information provided were cited as reasons for not seeking adequate informed consent during the COVID-19 pandemic [33,48]. Alternative consent procedures are in place for patients who are unable to consent. The patient who is unable to give consent is represented by his or her legal representative [23]. This procedure is in line with the Declaration of Helsinki [34]. A general exclusion of vulnerable patient groups from clinical studies without justification violates the self-determination of these patients. They should be given the opportunity to participate in COVID-19 studies following adequate informed consent [46]. It needs to be noted, however, that in some cases, patients may fear disadvantages if they refuse to participate in a clinical study when asked [24]. According to the Declaration of Helsinki, potential test subjects should not suffer any disadvantage due to their refusal to participate in a study [34]. Participation in a randomized, placebo-controlled study has an impact on the patient’s self-determination, as he or she cannot decide for or against a particular therapy. The study design does not allow the participant to actively choose to be treated with a particular experimental drug instead of being given a placebo. According to the Declaration of Helsinki, it is permissible to give a placebo if, as in the case of COVID-19, no proven intervention option is available [34,49].

### 3.4. Principle of Justice

The principle of justice demands fair distribution and fair access to health services [8]. A connection to the principle of justice can be found on four sub-themes: drug research, treatment outside of clinical trials, non-medical influences on drug use (e.g., political or economic interests), and distribution of limited medicinal products (including plasma). In *n* = 14 (26.9%) publications on hydroxychloroquine, *n* = 5 (9.6%) on remdesivir and *n* = 1 (1.9%) on COVID-19 convalescent plasma, the aspect of fairness in drug distribution was discussed. The assumption that hydroxychloroquine could be effective in COVID-19 led to supply shortages to treat patients for diseases for which the medicine was originally developed and approved [24,50]. This leads to a situation which is difficult to assess with the principle of justice. The interests of patients for which there is evidence that they will benefit from the drug need to be weighed against the interests of those patients for whom the drug is one of the few reasonable hopes for recovering under the state of emergency. This situation becomes even more difficult to evaluate from an ethical perspective, as there are no comparable drug alternatives for every approved indication [51]. Due to the increased demand, there are fears that the price of hydroxychloroquine will rise, making it inaccessible in low-income countries [26] and the uninsured. This involves the sub-theme “non-medical influences”. Not only medical but also financial aspects are decisive for the distribution and treatment with hydroxychloroquine. After the emergency use authorization was granted in the United States, not enough remdesivir was available for all eligible patients [37]. For both drugs, criteria had to be established for a fair distribution [37,50].

Participation in clinical trials is seen as an option to obtain potentially effective drugs on time [32]. Sometimes there are more interested participants for a clinical study than those that can be included in them [52]. Refusing participants in the study on paternalistic grounds contradicts the principle of respect for the patient’s autonomy. Although there is awareness that randomized studies are indispensable for identifying potential COVID-19 drugs and their use, these are frequently used in off-label use [49]. With off-label use, no scientifically reliable knowledge can be obtained about the drug used. As off-label use only occurs on a case-by-case basis, results cannot be generalized. There is also a risk of confirmation bias, as physicians may want to see a positive effect. Findings on the efficacy and safety of unsystematic off-label use studies have therefore little scientific value [33]. Despite the epistemic limitations of isolated experiences, a database that pools these different experiences together using quantifiable parameters may have scientific value on an aggregate level. One of the ethical recommendations after the experience from the SARS outbreak of 2003 is to promote data sharing practices [53]. During public health emergencies decisions often need to be taken with poor or no data. If clinicians abide to a duty to share data with centralized institutions, this data can be curated and be made publicly available to assist decision-making and thereby improve pandemic response capabilities.

During the early phase of the pandemic, some countries did not have enough patients to carry out clinical studies. If not enough patients are enrolled in clinical trials, it takes longer for effective treatments to be discovered and used [54]. When these trials are randomized, only a subset of patients will receive this medicine. With a view to treating patients equally, all patients are to be given the potentially effective drug [49]. Some authorities have now banned the use of hydroxychloroquine outside of clinical trials for COVID-19 [24]. The refusal to dispense hydroxychloroquine restricts self-determination in favor of harm avoidance and fair distribution [51]. Different types of drug distribution and selection criteria for inclusion in clinical trials to obtain drugs have been discussed. Possible strategies are distributions according to the best individual risk-benefit ratio, by prioritizing most health-endangered patients, or according to the “first come, first served” principle [37]. Researchers also see selection through a lottery system as an option [37,52]. When choosing participants for clinical trials, a selection criteria aspiring to increase the scientific value and the social benefit of the study can be ethically justified [52]. Making choices to mitigate or minimize the impact of existing health disparities has been discussed [15].

## 4. Discussion

### 4.1. The Adoption of Ethical Principles and Guidelines

Our results reveal a variety of ethical drug use challenges during the early phase of the COVID-19 pandemic. The availability of ethical guidelines from specialist societies facilitates ethical decision-making for medical staff. However, these were not initially available or needed adaptation to the new circumstances. The needed ethical guidelines were developed under great time pressure in the early phase of the pandemic, requiring further adjustments as the pandemic continued to reveal unforeseen social challenges, the pathogen developed new mutations and new scientific discoveries were made. These developments also lead to regular changes in recommended drug use. As there is still no regularly approved drug for the treatment of COVID-19 and satisfactory solutions for all ethical challenges have not been found, our findings from the early phase of the pandemic continue to offer important insights. Even after more than a year of a pandemic, it is necessary to continue to assess the ethical challenges that arose in the early phase of the pandemic and develop clear ethical guidelines based on this experience to mitigate current and future similar conflicts. For instance, we are currently witnessing similar ethical conflicts with COVID-19 vaccines. The unknown risks and the distribution of the limited number of vaccines available still raise major ethical questions. Our findings can help to initiate measures to prevent or mitigate ethical challenges in the further course of the pandemic. In addition, our findings are not only important for the ongoing pandemic but also can play a major role to prepare for similar outbreaks in the future [55].

We observed that ethical issues in connection with the use of drugs in clinical studies arise primarily in relation to informed consent and the design of the study. Weakening informed consent requirements contradicts the principle of respect for the patient’s autonomy but could be argued for by appealing to beneficence [8,33]. Demanding randomized trials during a pandemic is controversial [27,49,54]. Proponents claim that health crises are no excuse for lowering scientific standards [27]. Opponents defend the position that it is more important to treat patients in time based on current knowledge than to generate new knowledge following complex studies [54]. When it comes to questions of drug distribution, all four ethical principles collide [51]. A full consideration of the complex interactions between the ethical principles harbors ethical dilemmas for the treating physicians [24,49]. 

In our analysis of the experimental drug use during the early stage of the pandemic, the use of hydroxychloroquine was associated with the largest number of ethical conflicts, followed by remdesivir. COVID-19 convalescent plasma played a minor role in relation to hydroxychloroquine and remdesivir. The five sub-themes identified in the thematic analysis can be grouped in four discussion points by referring to the four ethical principles: Unknown risks and benefits ratios, informed consent, study design and fair distribution of drugs (Table 1).

### 4.2. The Adoption and Development of Ethical Principles and Guidelines in Germany

To illustrate the significance of these findings, we use the German regulatory system as a case study to discuss the extent to which the ethical controversies and recommendations identified in international publications are reflected in practice. We decided to focus on Germany for three reasons. First, all three analyzed medicinal products were among the main candidates for experimental treatment in the early phase of pandemic in Germany. According to the EU registry for clinical studies (as of 12 April 2021), all three drugs were used at some point during the early phase of the pandemic in clinical trials for COVID-19 in Germany. In addition, they were also used outside of clinical trials. None of the three drugs went through a regular drug approval process for COVID-19 in Germany [20,56]. Second, from early on, ethics groups were involved in providing ethical guidance on using drugs for experimental therapies. As different groups and institutions worked in parallel and in cooperation, we are able to refer to multiple position papers to illustrate how ethical guidelines became incorporated in public health policies and recommendations. Third, the work between ethics groups and public institutions was generally perceived as constructive and related publications were well received. Uniform guidelines were available early on nationwide, which was considered as desirable by other states. In Germany, the close collaboration between professional societies and the publishing of common guidelines prevented polarization. The Public Health Competence Network on COVID-19, for example, is an ad hoc association of more than 25 scientific specialist societies in the field of public health that bundle their specialist knowledge. Such associations could contribute to the rapid development of guidelines that enjoyed high acceptance and helped to support the stakeholders involved. Countries without standardized guidelines often had contradictory recommendations at regional and local levels. In some cases, medical professionals were not sure which guidelines to follow. Experts therefore called for uniform national ethical guidelines [57], such as those that were in use in Germany. 

#### 4.2.1. Unknown Risks and Benefits Ratios

Research results on hydroxychloroquine, remdesivir and COVID-19 convalescent plasma hold out the prospect of a potential benefit for people with COVID-19. During the early pandemic, the use of the three drugs on people with COVID-19 was compatible with the principle of beneficence, as COVID-19 was associated with a high mortality and hospitals were running out of capacity. At the same time, general clinical practice dictates cautiousness when convincing results from large clinical studies are missing. There is the risk that patients suffer greater harm than benefits when administered any of the three drugs, violating the principle of non-maleficence.

Remdesivir received conditional marketing authorization for the European Union member states on 3 July 2020. Before approval, it was available for compassionate use in Germany [20]. This formal change brought economic advantages for the pharmaceutical company, since drugs for use by patients in compassionate use in Germany have to be made available free of charge by the pharmaceutical manufacturer [10]. This is no longer the case with a conditional marketing authorization. The five-day treatment costs about 2.000 euros (2.340 dollars) per patient [58]. Even if recent data for remdesivir could not confirm a positive benefit-risk ratio, the company benefited financially during the period of conditional marketing authorization, which is limited to one year [18]. In the case of hydroxychloroquine, the influence of financial interests cannot be completely ignored either. Hydroxychloroquine is a relatively cheap drug at under 20 euros for 30 tablets in Germany. But scientists have already expressed concerns that the widespread promotion of hydroxychloroquine could encourage fraud and counterfeiting of drugs [26]. This can lead to additional risks. According to information from the Federal Institute for Drugs and Medical Devices (BfArM), COVID-19 patients in Germany can currently (April 2021) receive hydroxychloroquine in off-label use [20]. The use of COVID-19 convalescent plasma is permitted in Germany as a non-approved drug, outside of clinical studies, to carry out an individual healing attempt (Necessity as a justification, Section 34 German Criminal Code [22]). The Paul Ehrlich Institute published recommendations for its extraction and production [56]. Uncertainties regarding the potential benefits and harms of hydroxychloroquine, remdesivir and COVID-19 convalescent plasma are also recognized by German health authorities [20,56]. According to a survey by German ethics commissions, a risk-benefit assessment in intervention studies is problematic given the current state of knowledge [7].

#### 4.2.2. Informed Consent

According to Beauchamp and Childress, informed consent is the cornerstone of the principle of respect for autonomy and is therefore one of the most important processes in medical ethics [8]. The Declaration of Helsinki states that each patient must be adequately informed about the expected benefits and potential risks [34]. The provision of the necessary information to seek informed consent is difficult when risks and benefits are still not sufficiently known [32,48]. It must be made clear in the information process that the communicated risks and benefits involve uncertainties. Ideally, patients should be offered updated information as soon as new relevant scientific findings emerge to reassess their consent [59]. We identified ethical issues related to informed consent in the sub-themes “drug research” and “treatment outside of clinical trials” (Table 1). In Germany, ethical challenges in relation to informed consent concentrate on uncertainties with regard to alternative options for consent, the classification of patients as incapable of consenting and isolation measures [7]. In contrast to other scientists, the Working Group of the Medical Ethics Commissions in Germany does not consider the waiving of informed consent requirements for patients with an acute COVID-19 infection [4,33]. The Working Group supports the statements on informed consent of the “Guidance on the Management of Clinical Trials during the COVID-19 (Coronavirus) pandemic”, prepared under the coordination of the European Medicines Agency [4,60]. This guidance refers as an alternative to written consent by the trial participant, for example, by recognizing oral consent in the presence of an impartial witness or consent by a legal representative to minimize the spread of the disease [60]. The first version of this EU guidance was published on 20 March 2020 and thus offers the first ethical advice specifically tailored to COVID-19 at a very early stage of the pandemic. 

#### 4.2.3. Study Design

Germany was involved in studies on the use of hydroxychloroquine during the early phase of the pandemic. Currently, there is no reference to ongoing studies on hydroxychloroquine in COVID-19 with German participation in the EU Clinical Trials Register (as of 12 April 2021). Stopping a clinical trial with disappointing interim results can avoid harming patients and is in line with the principle of non-maleficence. Germany is still conducting clinical studies on remdesivir and COVID-19 convalescent plasma. There continues to be an expectation among scientists that these treatment options will deliver promising results. The use of remdesivir and COVID-19 convalescent plasma can therefore be in accordance with the principle of beneficence. The Working Group of the Medical Ethics Commissions in Germany points out that study results from COVID-19 research are needed quickly, but only qualitatively high-quality studies, such as interventional studies, can be rated positively as comparable controlled studies [4]. In this respect, German ethics commissions comply with the demand of not allowing lower scientific standards in research during the pandemic [27]. Germany is involved in the global drug study SOLIDARITY with a very heterogeneous study population. This study does not automatically exclude patient groups that are often neglected in research [12]. The automatic exclusion of specific groups contradicts the principle of respect for autonomy. However, in WHO registered studies within the “COVID-19 and pregnancy” category, no study was found whose sponsor is in Germany [45]. Since patients are better monitored in clinical trials than, for example, in compassionate use, participation in the study would be preferable for vulnerable groups and individuals in accordance with the principle of non-maleficence [10]. Ethics committees in Germany are also dealing with inclusion and exclusion criteria. For example, issues arise in the allocation of patients to studies when there are only a very limited number of infected people and they can be included in multiple studies at the same time [7]. Internationally, a differentiated picture emerges: there is sometimes an oversupply of studies, and sometimes an oversupply of potential study participants [27,52]. This situation has of course drastically changed since October 2020.

#### 4.2.4. Fair Distribution of Medicines

Although a shortfall of hydroxychloroquine is often mentioned in the literature, it does not contain any concrete figures of a supply bottleneck. It is reported that patients with *lupus erythematosus* were unable to redeem their prescriptions [51]. The actual extent of the shortfall is not specified. The situation in Germany seems to be comparable to the one depicted in international reports. In a survey at the end of April 2020 among *n* = 66 rheumatologists in Germany, 65.9% of those questioned stated that there was a shortage of the drugs they had prescribed. Hydroxychloroquine and chloroquine (*n* = 30) were mentioned most frequently [61]. The list of reported delivery bottlenecks of the BfArM did not reveal a delivery shortfall for hydroxychloroquine at any time. On 3 April 2020, the higher federal authority published a notice intended to ensure the supply of hydroxychloroquine for chronically ill patients with the approved indications [20]. A request to the BfArM for specific figures did not lead to any result. The action of the BfArM can be seen as an action in favor of patients who have already received hydroxychloroquine before the pandemic. It serves the principle of beneficence for these patients as they already have built their lives under the reasonable expectation that they can count with continuous access to the drug. Even if no serious ethical dilemmas due to drug shortages have been reported in Germany so far, this issue should continuously be reevaluated. If resources are scarce, the ethical prerequisites for permissible treatment measures set out in the guidelines on intensive care therapy would not be met. Medical indication and the patient’s will are not the only decisive factors [62]. With a view to the principle of justice, the interests of patients with prior conditions need to be taken in consideration when assessing issues of justice and should not be overseen during emergencies. Future pandemic planning needs to consider the difficult ethical question on how to proceed if scarce medicines used by people with prior conditions would actually save many more people when applied for newly identified uses.

#### 4.2.5. Institutionalization of Ethical Norms and Implications for Future Practice

During the pandemic, decisions often have to be made under time pressure. There is a constant temptation to override standard protocols to save time. However, in the analyzed publications, there is no call for limiting the involvement of ethics committees to save time. The crucial role of ethics committees during the pandemic in the assessment of ethical issues on drug use in clinical studies has been pointed out several times [30,32,33]. The regular participation of ethics committees in clinical studies in Germany, on the other hand, was restricted by Section 8 (2) of the “Medical Needs Supply Guarantee Ordinance” [63]. In contrast to the legal situation before the pandemic, a lead ethics commission evaluates multicenter clinical trials on COVID-19 in Germany, which are carried out in more than one trial center without further consultation with the local ethics committees. This legal change, with further ordinances and notices in the pharmaceutical sector, reduces regulatory hurdles during the pandemic. At first sight, the removal of regulatory hurdles to accelerate processes appears as beneficial, but it may lead to a failure to address the specific needs of those involved in drug use. There is consensus in Germany and at an international level that additional recommendations, guidelines and regulations for off-label use in crisis situations, drug distribution and clinical trials are useful [7,37,48,51,64]. The first concrete measures to develop appropriate recommendations have already been taken in Germany. As part of the PRECOPE project, practice-oriented recommendations for dealing with the most relevant ethical challenges in biomedical research using human subjects are being developed [7]. 

Comparable projects are missing for ethical questions regarding off-label use in crisis situations and fair distribution of drugs. Although there is an expert group for off-label use at the BfArM, it does not deal with ethical issues related to this [65]. A centralized registration of off-label use and its effects and side effects can facilitate future decision-making, especially if it includes uses without clinical trials, on the basis of small trials, or trials with study designs other than randomized controlled trials. Since off-label use not only plays a role in emergency situations but also in regular practice, especially in pediatrics and oncology, ethical recommendations should be drawn up for off-label use in and outside of emergency. To make sure physicians share crucial data even under time pressure, ethicist need to provide strong arguments on why data sharing is ethically demanded by ideas of reciprocity and solidarity [53]. 

In line with the principle of justice, considerations should be made for the equitable distribution of medicines. Professional societies are currently preparing a recommendation for the appropriate use of remdesivir. This should lead to guaranteed access for patients who could benefit from remdesivir [66]. In addition to questions of proper use, we face ethical challenges when there are insufficient drugs available for all eligible patients. Making recommendations for the distribution of limited medical supplies poses many ethical challenges [2]. 

### 4.3. Summary of General Recommendations

Future recommendations need to be drawn up at an early stage and made publicly available. They should not be drafted under time pressure and already discuss a wide range of possible scenarios. We have listed our main findings from our ethical analysis in Table 2. 

### 4.4. Limitations

Our analysis concentrates on the first ten months of the pandemic to identify the main ethical arguments on experimental medicines that acted as guiding principles during the early phase of the COVID-19 outbreak. Extensive ongoing research activity on COVID-19 comes with a rapid change in the state of knowledge on pharmaceutical treatment options. For example, a year after the start of the pandemic, hydroxychloroquine no longer plays a major role as a treatment option for COVID-19 [67]. In contrast, other drugs such as dexamethasone, the antibodies bamlanivimab and RegnCoV-2 or the asthma spray budenoside are gaining attention in research and medical practice [20,68,69]. The use of many of these new drugs follows the same ethical reasoning behind our analyzed cases on experimental drug use. Therefore, while the drugs being used experimentally have changed, the ethical challenges identified in our analysis are still similar even after more than a year of COVID-19 pandemic. This ethical analysis is widely transferable to other potentially effective medications during public health emergencies and can assist ethical decision-making in future pandemic planning. At the time of writing (April 2021), there are no medicines that have shown fully supportive evidence in a phase 3 trial and have gone through a regular authorization procedure.

A shortcoming of having relied on a principle-based approach, is that such an ethical approach is primarily forward-looking. This approach has the benefit of providing clear guidelines with which affected groups and policymakers can familiarize themselves. While the principle of justice allows us to identify ethical issues that need to be redressed, it does not emphasize the importance of continuously carrying out an ethical assessment when encountering new information to revise guidelines. Future work on experimental drug uses needs to explore the potential of ethical approaches that have a solid backward-looking component, who place a high value on “reasonableness” and “responsiveness” [40] to continuously revise guidelines when encountering new problems and opportunities. 

## 5. Conclusions

This analysis of the ethical challenges in relation to drug use during the early COVID-19 pandemic shows that drug safety and distribution issues affect all four ethical principles, leading to frequent conflicts between them. Among the main ethical challenges we have identified, we discussed the effect of unknown risks and benefits, informed consent, study design limitations and the fair distribution of medicines. Drug safety issues are equally challenging for all three hydroxychloroquine, remdesivir and COVID-19 convalescent plasma. In contrast to the international discussion, the fair distribution of medicines seems to play a subordinate role in Germany. Only hydroxychloroquine briefly came into the focus of ethical discussions. Our results confirm some of the ethical challenges addressed in the PRECOPE project. In relation to the ethical aspects of drug use, they go beyond this, as they also include their experimental use outside of clinical studies. Knowledge of these challenges offers the opportunity to take appropriate action. The drafting and implementation of new ethical recommendations can help to minimize ethical challenges and thereby lead to positive effects on future clinical research, the administration of drugs in off-label use and drug distribution in and outside of times of emergency.

## Figures and Tables

**Table 1:** Determining ethical arguments within the five identified sub-themes.

Identified Sub-Themes	Principles Involved According to Beauchamp and Childress	Determining Ethical Arguments
Existing data on pharmacological treatment	Beneficence Non-maleficence	Unknown risks and benefits ratios
Drug research	Beneficence Non-maleficence Autonomy Justice	Unknown risks and benefits ratios Informed consent Study design
Treatment outside of clinical trials	Beneficence Non-maleficence Autonomy Justice	Unknown risks and benefits ratios Informed consent Fair distribution of drugs
Non-medical influences on drug use (e.g., political or economic interests)	Non-maleficence Justice	Unknown risks and benefits ratios Fair distribution of drugs
Distribution of limited medicinal products (including plasma)	Beneficence Non-maleficence Autonomy Justice	Unknown risks and benefits ratios Fair distribution of drugs

**Table 2:** Recommendations based on the experience during the early pandemic.

Ethical Principle	Recommendations
Beneficence	- Maintain openness to discuss experimental treatment options with patients from all population groups who are in urgent need
Non-maleficence	- Expand inclusion for clinical studies criteria to avoid missing potentially negative effects on excluded groups (e.g., pregnant women, elderly)
Respect for autonomy	- Avoid paternalistic attitudes when excluding systematically participants from higher risks groups
Justice	- Establish guidelines to secure sufficient access to repurposed drugs for patients with prior conditions - Establish protocols to authorize the recording of patient reactions to drugs in off-label use

## Data Availability

The data presented in this study are available within the article.
